# Effect of preoperative fasting on acute postoperative pain following cesarean section: a retrospective cohort study

**DOI:** 10.1097/MS9.0000000000004865

**Published:** 2026-04-14

**Authors:** Huiyan Xu, Yanshuang Wang, Dongge Niu, MingJun Xu, Lan Yao

**Affiliations:** a Department of Anesthesiology, Peking University International Hospital, Beijing, China; b Department of Anesthesiology, Beijing Obstetrics and Gynecology Hospital, Capital Medical University, Beijing Maternal and Child Health Care Hospital, Beijing, China

**Keywords:** cesarean section, ERAS, postoperative pain, preoperative fasting, VAS

## Abstract

**Background:**

Preoperative fasting is a cornerstone of Enhanced Recovery After Surgery (ERAS) protocols in cesarean delivery, primarily aimed at reducing aspiration risk. However, its impact on acute postoperative pain remains unclear. This study addresses a critical evidence gap regarding whether adherence to ERAS-compliant fasting guidelines influences post-cesarean pain intensity.

**Methods:**

Data from 329 women who underwent cesarean sections at the hospital between March and December 2024 were retrospectively reviewed. Women were categorized based on adherence to ERAS fasting guidelines (≤8 h solids, ≤2 h clear fluids). The primary outcome was the 24-h postoperative Visual Analogue Scale (VAS) pain score. Univariate and multivariable linear regression models were used to assess the association between fasting compliance and VAS scores, adjusting for key covariates including age, body mass index, comorbidities, anesthesia type, surgical duration, and intraoperative morphine use.

**Results:**

Women adhering to ERAS fasting protocols had significantly lower mean 24-h VAS scores compared to non-adherent women (β = −0.6; 95% confidence interval: −1.2, −0.1; *P* = 0.029). This association remained significant after minor (β = −0.6; *P* = 0.046) and partial adjustment (β = −0.6; *P* = 0.038), though it attenuated to non-significance after full adjustment including epidural morphine (β = −0.5; *P* = 0.137). Subgroup analyses suggested consistent directional effects, but detailed subgroup results are reported in the main text.

**Conclusions:**

Adherence to ERAS fasting guidelines is associated with a modest but statistically significant reduction in acute postoperative pain after cesarean section. While the clinical relevance of a 0.6-point VAS difference may be limited, these findings support integrating standardized preoperative fasting into obstetric ERAS pathways as a safe, low-cost component of multimodal pain management and enhanced recovery.

## Introduction

Cesarean section is a common and often life-saving obstetric intervention, yet optimal perioperative management remains essential to enhance maternal outcomes^[^1–4^]^. Preoperative fasting – traditionally defined as abstaining from solids for 6–8 h and clear fluids for 2 h before surgery – is widely practiced to reduce gastric volume and mitigate aspiration risk. Current Enhanced Recovery After Surgery (ERAS) guidelines endorse these shorter fasting intervals as safe and beneficial in obstetric populations.

Despite broad adherence to ERAS-compliant fasting protocols, limited evidence exists on their impact on postoperative pain following cesarean delivery. While fasting is primarily aimed at preventing pulmonary complications, its potential influence on pain intensity – an important component of recovery – has not been systematically evaluated.

This study hypothesizes that adherence to ERAS fasting guidelines is associated with reduced postoperative pain intensity. We conducted a retrospective cohort analysis of women undergoing cesarean section at our institution to assess whether compliance with recommended fasting durations (≤8 h solids, ≤2 h clear fluids) correlates with lower 24-h postoperative Visual Analog Scale (VAS) pain scores and improved analgesic outcomes. By addressing this knowledge gap, our findings aim to inform evidence-based preoperative strategies that support both safety and comfort in cesarean delivery.

## Methods and materials

**Figure 1.**
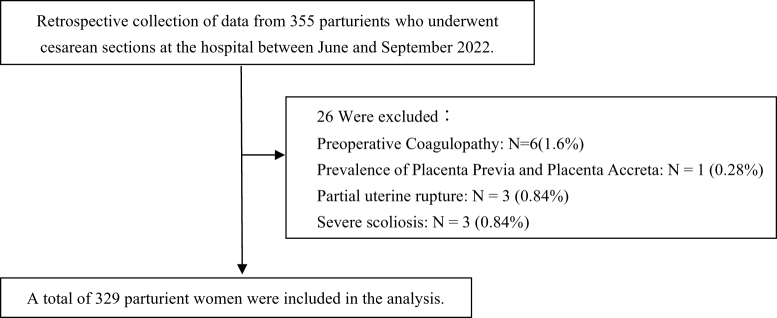
Flow chart of study population.

We conducted a retrospective review of data collected from 355 women who underwent cesarean sections at the hospital between March and December 2024 as demonstrated in Fig. 1. All pregnant women provided informed consent for anesthesia prior to surgery. This study was approved by the Biomedical Ethics Committee of Peking University International Hospital (Approval No.: 2024-KY-0003-02). The requirement for informed consent was waived due to the retrospective nature of the study and the use of anonymized clinical data. All procedures were conducted in accordance with the ethical standards of the Declaration of Helsinki. To ensure data accuracy, two independent researchers extracted all variables from electronic medical records using a standardized form. Discrepancies were resolved by consensus or adjudication by a third reviewer. Key variables (e.g., fasting duration, VAS scores, morphine dose) were cross-verified against anesthesia and nursing documentation. This cohort included women who utilized patient-controlled epidural analgesia (PCEA) pumps postoperatively, covering cases ranging from those who had cesarean sections following vaginal delivery attempts to those who underwent spinal anesthesia for elective cesarean sections. The research has been presented following the STROCSS criteria^[^5^]^.

Inclusion criteria were as folllows: (1) The preoperative assessment records of women must strictly adhere to the classification system of the American Society of Anesthesiologists (ASA). (2) The clinical medical records of the research subjects need to be complete and standardized. (3) The women incorporated into the study must have full-term deliveries, with the gestational age reaching or exceeding 37 weeks. (4) The selected cases of cesarean section must conform to the surgical indications for cesarean section formulated by the Obstetrics Group of the Obstetrics and Gynecology Branch of the Chinese Medical Association. (5) All the women included in the study must undergo the same anesthesia method, namely combined spinal-epidural anesthesia.

Postpartum pain following surgery was assessed within 24 h using the VAS.

### Statistical analysis

Continuous variables were expressed as median (min–max) or mean ± standard deviation, depending on the data distribution. Categorical variables were expressed as frequencies or percentages. The Mann–Whitney and Chi-square tests were used for continuous and categorical variables, respectively. Unadjusted and adjusted linear regression β coefficients with corresponding 95% confidence intervals (CIs) were estimated and reported to quantify the association between preoperative fasting adherence and 24-h postoperative VAS scores (see Table 1). All data analyses were performed using the statistical software packages R (http://www.R-project.org, The R Foundation) and EmpowerStats (http://www.empowerstats.com, X&Y Solutions, Inc., Boston, MA). *P* values <0.05 (two-sided) were considered statistically significant.

HIGHLIGHTS

- **Adherence to the eras protocol fasting guidelines improves analgesia**
- It’s been shown that following the ERAS (Accelerated Rehabilitation Surgery) recommended preoperative fasting time (6–8 h for solid foods and 2 h for liquids) significantly improves analgesia within 24 h after cesarean section, reduces pain scores and optimizes maternal recovery after the operation.

- **Fasting regulates the inflammatory response to reduce pain**
- Appropriate preoperative fasting reduces the inflammatory response by regulating the inflammatory mediators in the body, which helps to reduce the intensity of postoperative pain and promotes the rapid return of gastrointestinal function to normal, thus reducing the feeling of abdominal distension and discomfort.

- **Caution in reducing the duration of preoperative water fasting**
- Although reducing the duration of preoperative fasting may improve patient comfort, recommendations to reduce the duration of preoperative water fasting need to be approached with caution to avoid increasing the risk of gastroesophageal reflux and aspiration during anesthesia

## Results

Baseline characteristics of the women are presented in Table 1.

**Table 1:** Baseline characteristics of participants.

Preoperative fasting	No	Yes	Standardize diff.	*P*-value
Variables	143	186		
Age (years)	33.34 ± 4.01	34.17 ± 4.38	0.20 (−0.02, 0.42)	0.079
Height (cm)	162.4 ± 5.5	161.8 ± 5.3	0.1 (−0.1, 0.3)	0.299
Weight (kg)	74.1 ± 11.2	75.2 ± 11.6	0.1 (−0.1, 0.3)	0.394
Body mass index	28.1 ± 3.8	28.7 ± 4.0	0.2 (−0.1, 0.4)	0.153
Intraoperative blood loss (continuous)	449.1 ± 128.1	416.5 ± 119.6	0.3 (0.0, 0.5)	0.018
Intraoperative rehydration continuous	829.4 ± 165.7	837.3 ± 194.7	0.0 (−0.2, 0.3)	0.697
ASA			0.1 (−0.1, 0.3)	0.283
II	132 (92.3%)	177 (95.2%)		
III	11 (7.7%)	9 (4.8%)		
Different ratios of sufentanil and ropivacaine			0.5 (0.3, 0.8)	0.097
Non-utilization of PCEA	12 (8.4%)	5 (2.7%)		
75 mg of ropivacaine and no sufentanil (0 μg).”	1 (0.7%)	0 (0.0%)		
25 µg of sufentanil and no ropivacaine (0 mg).	4 (2.8%)	7 (3.8%)		
25 µg of sufentanil and 100 mg ropivacaine	62 (43.4%)	91 (48.9%)		
25 µg of sufentanil and 150 mg ropivacaine	1 (0.7%)	0 (0.0%)		
25 µg of sufentanil and 50 mg ropivacaine	1 (0.7%)	6 (3.2%)		
25 µg of sufentanil and 60 mg ropivacaine	1 (0.7%)	0 (0.0%)		
25 µg of sufentanil and 75 mg ropivacaine	48 (33.6%)	72 (38.7%)		
25 µg of sufentanil and 80 mg ropivacaine	1 (0.7%)	0 (0.0%)		
30 µg of sufentanil and 100 mg ropivacaine	1 (0.7%)	0 (0.0%)		
30 µg of sufentanil and 75 mg ropivacaine	2 (1.4%)	0 (0.0%)		
30 µg of sufentanil and no ropivacaine (0 mg).	1 (0.7%)	0 (0.0%)		
35 µg of sufentanil and 100 mg ropivacaine	0 (0.0%)	1 (0.5%)		
40 µg of sufentanil and 100 mg ropivacaine	2 (1.4%)	1 (0.5%)		
50 µg of sufentanil and no ropivacaine (0 mg).	3 (2.1%)	1 (0.5%)		
50 µg of sufentanil and 100 mg ropivacaine	2 (1.4%)	2 (1.1%)		
50 µg of sufentanil and 50 mg ropivacaine	1 (0.7%)	0 (0.0%)		
Use of PCEA			0.3 (0.0, 0.5)	0.021
No	12 (8.4%)	5 (2.7%)		
Yes	131 (91.6%)	181 (97.3%)		
Ropivacaine dosage in PCEA (continuous)	76.7 ± 33.2	81.2 ± 26.4	0.2 (−0.1, 0.4)	0.166
Sufentanil dosage in PCEA (continuous)	24.0 ± 9.5	24.9 ± 5.6	0.1 (−0.1, 0.3)	0.255
Hypertension in pregnancy			0.1 (−0.1, 0.3)	0.572
Healthy	133 (93.0%)	171 (91.9%)		
With hypertension	8 (5.6%)	14 (7.5%)		
With severe preeclampsia	2 (1.4%)	1 (0.5%)		
Epidural morphine administration (mg)			0.5 (0.3, 0.7)	<0.001
0	67 (46.9%)	58 (31.2%)		
1	3 (2.1%)	0 (0.0%)		
1.2	0 (0.0%)	3 (1.6%)		
1.5	8 (5.6%)	3 (1.6%)		
2	65 (45.5%)	122 (65.6%)		
Epidural morphine administration(mg) (continuous)	1.0 ± 1.0	1.4 ± 0.9	0.4 (0.1, 0.6)	0.001
Gestational diabetes			0.2 (−0.0, 0.4)	0.298
Pregnancy complicated with ketosis	3 (2.1%)	2 (1.1%)		
Gestational diabetes mellitus	20 (14.0%)	37 (19.9%)		
Healthy	120 (83.9%)	147 (79.0%)		
Preoperative fasting				<0.001
No	143 (100.0%)	0 (0.0%)		
Yes	0 (0.0%)	186 (100.0%)		
VAS			0.4 (0.2, 0.6)	0.211
0	7 (4.9%)	5 (2.7%)		
1	3 (2.1%)	8 (4.3%)		
2	8 (5.6%)	12 (6.5%)		
3	6 (4.2%)	16 (8.6%)		
4	8 (5.6%)	19 (10.2%)		
5	19 (13.3%)	35 (18.8%)		
6	16 (11.2%)	21 (11.3%)		
7	30 (21.0%)	26 (14.0%)		
8	23 (16.1%)	25 (13.4%)		
9	9 (6.3%)	7 (3.8%)		
10	14 (9.8%)	12 (6.5%)		
VAS continuous	6.1 ± 2.6	5.5 ± 2.5	0.2 (0.0, 0.5)	0.029

OR, odds ratio, CI, confidence interval, β, regression coefficient, VAS, Visual Analog Scale.

In the study cohort of 329 women, 309 cases (93.9%) were classified as ASA II, while 20 cases (6.1%) were categorized as ASA III, with an average age of 33.81 ± 4.24 years. Of the total cases, 143 individuals adhered to the ERAS fasting protocol, whereas 186 individuals did not. The participants were categorized accordingly, and Table 2 illustrates the distribution of various populations for each indicator within the two groups. Postoperative acute VAS, epidural morphine dosage, intraoperative fluid infusion volume, and blood loss were treated as continuous variables. Postoperative acute VAS, epidural morphine dosage, intraoperative fluid infusion volume, and blood loss were treated as continuous variables.

**Table 2:** Univariate analysis of impact of each indicator on VAS pain score.

	Statistics	P1 continuous
ASA		
II	309 (93.9%)	0
III	20 (6.1%)	1.5 (0.4, 2.7) 0.010
Height (cm)	162.0 ± 5.4	0.0 (−0.0, 0.1) 0.148
Weight (kg)	74.7 ± 11.5	−0.0 (−0.0, 0.0) 0.862
Age (years)	33.8 ± 4.2	0.0 (−0.0, 0.1) 0.424
Preoperative fasting		
No	143 (43.5%)	0
Yes	186 (56.5%)	−0.6 (−1.2, −0.1) 0.029
Hypertension in pregnancy		
Healthy	304 (92.4%)	0
With hypertension	22 (6.7%)	−0.7 (−1.8, 0.5) 0.247
With severe preeclampsia	3 (0.9%)	1.5 (−1.4, 4.5) 0.304
Gestational diabetes		
Pregnancy complicated with ketosis	5 (1.5%)	0
Gestational diabetes mellitus	57 (17.3%)	−1.1 (−3.5, 1.2) 0.347
Healthy	267 (81.2%)	−1.0 (−3.3, 1.3) 0.376
Epidural morphine administration (mg)		
0	125 (38.0%)	0
1	3 (0.9%)	−0.6 (−3.5, 2.4) 0.709
1.2	3 (0.9%)	−0.9 (−3.8, 2.0) 0.551
1.5	11 (3.3%)	0.5 (−1.1, 2.1) 0.532
2	187 (56.8%)	−0.8 (−1.4, −0.2) 0.006
Epidural morphine administration (mg) (continuous)	1.2 ± 1.0	−0.4 (−0.7, −0.1) 0.008
Different ratios of sufentanil and ropivacaine		
Non-utilization of PCEA	17 (5.2%)	0
75 mg of ropivacaine and no sufentanil (0 μg).”	1 (0.3%)	−0.5 (−5.7, 4.6) 0.841
25 μg of sufentanil and no ropivacaine (0 mg).	11 (3.3%)	0.3 (−1.6, 2.2) 0.770
25 μg of sufentanil and 100 mg ropivacaine	153 (46.5%)	0.4 (−0.9, 1.6) 0.576
25 μg of sufentanil and 150 mg ropivacaine	1 (0.3%)	0.5 (−4.7, 5.6) 0.858
25 μg of sufentanil and 50 mg ropivacaine	7 (2.1%)	−0.7 (−2.9, 1.6) 0.558
25 μg of sufentanil and 60 mg ropivacaine	1 (0.3%)	−1.5 (−6.7, 3.6) 0.561
25 μg of sufentanil and 75 mg ropivacaine	120 (36.5%)	0.3 (−1.0, 1.6) 0.629
25 μg of sufentanil and 80 mg ropivacaine	1 (0.3%)	1.5 (−3.7, 6.6) 0.576
30 μg of sufentanil and 100 mg ropivacaine	1 (0.3%)	0.5 (−4.7, 5.6) 0.858
30 μg of sufentanil and 75 mg ropivacaine	2 (0.6%)	2.5 (−1.3, 6.2) 0.197
30 μg of sufentanil and no ropivacaine (0 mg).	1 (0.3%)	0.5 (−4.7, 5.6) 0.858
35 μg of sufentanil and 100 mg ropivacaine	1 (0.3%)	−4.5 (−9.7, 0.6) 0.086
40 μg of sufentanil and 100 mg ropivacaine	3 (0.9%)	2.1 (−1.0, 5.3) 0.183
50 μg of sufentanil and no ropivacaine (0 mg).	4 (1.2%)	−1.8 (−4.6, 1.0) 0.211
50 μg of sufentanil and 100 mg ropivacaine	4 (1.2%)	−2.5 (−5.3, 0.3) 0.076
50 μg of sufentanil and 50 mg ropivacaine	1 (0.3%)	−5.5 (−10.7, −0.4) 0.036
Use of PCEA		
No	17 (5.2%)	0
Yes	312 (94.8%)	0.2 (−1.0, 1.5) 0.698
Intraoperative blood loss (ml) (continuous)	833.8 ± 182.4	0.0 (−0.0, 0.0) 0.090
Intraoperative rehydration (ml) (continuous)	430.7 ± 124.3	0.0 (−0.0, 0.0) 0.392

In univariate analysis presented in Table 3, it was found that women complied with the ERAS fasting protocol had lower postoperative VAS scores (−0.6; 95% CI −1.2, −0.1; *P* = 0.029) compared to those who did not. The use of epidural morphine showed a significant reduction in VAS scores (continuous variable) by −0.4 (−0.7, −0.1), *P* = 0.008. In practical terms, among 187 women treated with 2 mg epidural morphine, the VAS score effect was −0.8 (−1.4, −0.2), *P* = 0.006. Among women who fasted, ASA III women had higher postoperative VAS scores, while in women who did not fast. Age, body mass index, PCEA use, intraoperative fluid infusion, and blood loss did not show a significant association with perioperative VAS score reduction.

**Table 3:** Stratified analysis demonstrated the association between preoperative fasting and postoperative acute VAS across different subgroups.

	Preoperative fasting = No	Preoperative fasting = Yes	Total
Hypertension in pregnancy			
Healthy	0	0	0
With hypertension	1.3 (−0.3, 3.0) 0.103	1.6 (−0.1, 3.2) 0.064	1.5 (0.3, 2.6) 0.014
With severe preeclampsia	0.1 (−0.0, 0.1) 0.216	0.0 (−0.0, 0.1) 0.512	0.0 (−0.0, 0.1) 0.184
Gestational diabetes	−0.0 (−0.0, 0.0) 0.841	0.0 (−0.0, 0.0) 0.935	−0.0 (−0.0, 0.0) 0.942
Pregnancy complicated with ketosis	0.1 (−0.0, 0.2) 0.175	0.0 (−0.1, 0.1) 0.846	0.0 (−0.0, 0.1) 0.307
Gestational diabetes mellitus			
Healthy	0	0	0
Epidural morphine administration(mg)	0.6 (−1.3, 2.4) 0.558	−1.3 (−2.7, 0.1) 0.061	−0.6 (−1.7, 0.5) 0.278
0	1.9 (−1.7, 5.6) 0.304	0.4 (−4.5, 5.3) 0.870	1.4 (−1.5, 4.3) 0.347
1			
1.2	0	0	0
1.5	−0.1 (−3.3, 3.1) 0.968	−1.8 (−5.4, 1.7) 0.314	−1.0 (−3.3, 1.4) 0.414
2	−0.6 (−3.7, 2.4) 0.679	−1.4 (−4.9, 2.1) 0.420	−0.9 (−3.2, 1.3) 0.418
Epidural morphine administration (mg) (continuous)			
Different ratios of sufentanil and ropivacaine	0	0	0
Non-utilization of PCEA	−0.8 (−3.9, 2.2) 0.585	NA	−0.8 (−3.7, 2.2) 0.605
75 mg of ropivacaine and no sufentanil (0 μg).”	NA	−0.6 (−3.5, 2.3) 0.705	−0.6 (−3.6, 2.3) 0.670
25 µg of sufentanil and no ropivacaine (0 mg).	0.9 (−1.0, 2.8) 0.375	−0.9 (−3.8, 2.0) 0.547	0.4 (−1.2, 2.0) 0.607
25 µg of sufentanil and 100 mg ropivacaine	−0.9 (−1.8, −0.0) 0.044	−0.6 (−1.4, 0.2) 0.151	−0.7 (−1.3, −0.1) 0.016
25 µg of sufentanil and 150 mg ropivacaine	−0.4 (−0.9, 0.0) 0.067	−0.3 (−0.7, 0.1) 0.148	−0.3 (−0.6, −0.1) 0.020
25 µg of sufentanil and 50 mg ropivacaine			
25 µg of sufentanil and 60 mg ropivacaine	0	0	0
25 µg of sufentanil and 75 mg ropivacaine	−0.5 (−6.0, 5.0) 0.858	NA	−0.7 (−5.9, 4.4) 0.780
25 µg of sufentanil and 80 mg ropivacaine	1.3 (−1.8, 4.3) 0.421	−0.3 (−3.1, 2.5) 0.825	0.5 (−1.4, 2.5) 0.596
30 µg of sufentanil and 100 mg ropivacaine	0.6 (−1.0, 2.3) 0.458	0.1 (−2.0, 2.3) 0.903	0.6 (−0.7, 1.9) 0.382
30 µg of sufentanil and 75 mg ropivacaine	0.5 (−5.0, 6.0) 0.858	NA	0.3 (−4.9, 5.4) 0.918
30 µg of sufentanil and no ropivacaine (0 mg).	2.5 (−3.0, 8.0) 0.372	−1.3 (−4.1, 1.6) 0.389	−0.3 (−2.5, 2.0) 0.805
35 µg of sufentanil and 100 mg ropivacaine	−1.5 (−7.0, 4.0) 0.592	NA	−1.7 (−6.9, 3.4) 0.508
40 µg of sufentanil and 100 mg ropivacaine	0.8 (−0.9, 2.5) 0.349	−0.1 (−2.3, 2.1) 0.959	0.5 (−0.8, 1.8) 0.424
50 µg of sufentanil and no ropivacaine (0 mg).	1.5 (−4.0, 7.0) 0.592	NA	1.3 (−3.9, 6.4) 0.628
50 µg of sufentanil and 100 mg ropivacaine	0.5 (−5.0, 6.0) 0.858	NA	0.3 (−4.9, 5.4) 0.918
50 µg of sufentanil and 50 mg ropivacaine	2.5 (−1.5, 6.5) 0.224	NA	2.3 (−1.5, 6.0) 0.233
Hypertension in pregnancy	0.5 (−5.0, 6.0) 0.858	NA	0.3 (−4.9, 5.4) 0.918
Healthy	NA	−4.6 (−9.8, 0.6) 0.085	−4.0 (−9.2, 1.1) 0.124
With hypertension	2.0 (−2.0, 6.0) 0.330	2.4 (−2.8, 7.6) 0.367	2.2 (−1.0, 5.3) 0.174
With severe preeclampsia	−0.8 (−4.2, 2.6) 0.631	−4.6 (−9.8, 0.6) 0.085	−1.8 (−4.6, 1.0) 0.200
Gestational diabetes	0.5 (−3.5, 4.5) 0.807	−5.6 (−9.6, −1.6) 0.006	−2.4 (−5.2, 0.4) 0.092
Pregnancy complicated with ketosis	−5.5 (−11.0, −0.0) 0.051	NA	−5.7 (−10.9, −0.6) 0.029
Use of PCEA			
No	0	0	0
Yes	0.7 (−0.9, 2.2) 0.397	−0.1 (−2.3, 2.1) 0.924	0.4 (−0.8, 1.7) 0.500
Intraoperative blood loss (ml) (continuous)	0.0 (−0.0, 0.0) 0.579	0.0 (−0.0, 0.0) 0.071	0.0 (−0.0, 0.0) 0.079
Intraoperative rehydration (ml) (continuous)	0.0 (−0.0, 0.0) 0.435	0.0 (−0.0, 0.0) 0.967	0.0 (−0.0, 0.0) 0.561

Stratified analysis shown in Table 4 demonstrated the association between preoperative fasting and postoperative acute VAS across different subgroups: ASA II −0.6 (−1.2, −0.0) 0.0447, height −0.9 (−1.8, −0.0) 0.0466, age −0.9 (−1.8, −0.0) 0.0427, diabetes alone −1.4 (−2.8, −0.1) 0.0378, hypertension alone −2.3 (−4.2, −0.5) 0.0236, low intraoperative fluid infusion −1.3 (−2.4, −0.1) 0.0302, high intraoperative bleeding −0.9 (−1.5, −0.2) 0.0085. Additionally, stabilization of effect sizes was observed in other subcategories.

**Table 4:** Multiple regressions reveal a significant correlation between following the ERAS fasting protocol and a decrease in acute postoperative VAS scores.

Exposure	Adjust I	Adjust II	Adjust III
Preoperative fasting			
No	0	0	0
Yes	−0.6 (−1.1, −0.0) 0.046	−0.6 (−1.2, −0.0) 0.038	−0.5 (−1.0, 0.1) 0.137

Variables: β (95% CI) *P*-value/OR (95% CI) *P*-value.

Outcomes: VAS continuous.

Exposure: Preoperative fasting.

Adjust I model adjust for: ASA; height; weight; age.

Adjust II model adjust for: ASA; height; weight; age; hypertension in pregnancy; gestational diabetes; Different ratios of sufentanil and ropivacaine; intraoperative rehydration continuous; postpartum bleeding continuous.

Adjust III model adjust for: ASA; height; weight; age; hypertension in pregnancy; gestational diabetes; different ratios of sufentanil and ropivacaine; intraoperative rehydration continuous; postpartum bleeding continuous; morphine.

Multiple regressions illustrated in Table 1 revealed a significant correlation between following the ERAS fasting protocol and a decrease in acute postoperative VAS scores. The results remain stable with minor adjustments −0.6 (−1.1, −0.0) 0.046 or partial adjustments −0.6 (−1.2, −0.0) 0.038. However, when making full adjustments by adding morphine, although the significance diminishes, the effect size remains consistent −0.5 (−1.0, 0.1) 0.137.

## Discussion

When exploring the impact of preoperative fasting times on women undergoing cesarean delivery, we must carefully weigh the potential risks and benefits of reducing this period^[^6–9^]^.

While some studies suggest that shortening the preoperative fasting period may enhance maternal comfort, this approach remains controversial. The primary concern revolves around the risk of gastroesophageal reflux and aspiration during anesthesia. Notably, adhering to appropriate preoperative fasting guidelines not only helps mitigate these risks but may also indirectly promote better postoperative pain control and recovery^[^10–13^]^.

This study delves into the relationship between preoperative fasting strategies for cesarean sections (solid food fasting for 6–8 h and liquid fasting for 2 h) and postoperative acute pain management outcomes. It was found that adherence to the fasting guidelines, defined as “fasting yes,” was significantly associated with improved analgesic effects within the first 24 h post-surgery. This finding not only validates the effectiveness of current preoperative fasting guidelines but also provides strong evidence for optimizing preoperative preparation protocols for cesarean deliveries.

### Effect of preoperative fasting duration on postoperative pain in cesarean sections

Through univariate and stratified analyses, this study revealed a significant association between adherence to ERAS guidelines for fasting and postoperative acute pain management following cesarean section. Secondary outcomes included reductions in opioid consumption. ERAC is associated with superior maternal outcomes including decreased length of hospital stay, opioid consumption, pain scores^[^14,15^]^. After conducting sensitivity analysis tests, the results were found to be stable. Nevertheless, certain studies propose that there is no relationship between decreased opioid consumption and diminished peak postoperative pain.

A significant difference in opioid consumption and in per-day postoperative opioid consumption, lower peak pain scores were found after the introduction of the ERAS protocol. The reduced opioid use was not associated with an increase in pain scores; indeed, the peak pain scores were lower in the ERAS group. Our results highlight the importance of a scheduled non-opioid regimen for post-CD pain management^[^15,16^]^. In addition to our multimodal pain management strategy, other factors may have contributed. Patient anxiety, anticipated pain and need for analgesics correlate with pain intensity following CD^[^17^]^.

Our findings align with recent ERAS-focused cesarean studies. Ciechanowicz *et al*^[^14^]^ reported that full ERAS bundle implementation – including liberalized fluid intake – was associated with reduced opioid consumption and earlier mobilization, though pain scores were not significantly different. Similarly, Pinho and Costa^[^15^]^ observed no major VAS differences but noted improved patient satisfaction with abbreviated fasting. In contrast, our data suggest a small but measurable analgesic benefit specifically linked to fasting compliance, reinforcing its potential role within a comprehensive ERAS pathway – even if not transformative in isolation.

Importantly, subgroup analyses should be interpreted cautiously. Apparent greater benefits among ASA III patients or those with diabetes/hypertension may reflect residual confounding or chance findings given the limited sample size in these strata. These signals warrant hypothesis-generating exploration in future prospective trials but do not justify targeted prolonged fasting outside controlled settings.

Fasting may contribute to modest reductions in early postoperative pain through several hypothetical physiological pathways. Preclinical and limited clinical evidence suggests that short-term fasting could attenuate the surgical stress response and modulate inflammatory mediators [e.g., interleukin-6 (IL-6), cortisol], potentially dampening central sensitization to pain in women^[^16^]^. Additionally, by promoting earlier return of gastrointestinal motility, appropriate preoperative fasting might reduce postoperative abdominal distension and visceral discomfort^[^17,18^]^ – factors that can exacerbate somatic pain perception. However, it is important to note that the primary rationale for preoperative fasting remains the prevention of pulmonary aspiration during anesthesia^[^19^]^; any analgesic benefit is likely secondary and not directly causally linked to gastric emptying or anesthesia safety. The observed association between ERAS-compliant fasting and lower VAS scores in our study may therefore reflect either a direct biological effect, improved protocol adherence as a marker of overall care quality, or residual confounding. Further studies with biomarker integration are needed to test these mechanistic hypotheses.

### Role of epidural morphine in postoperative pain management

Univariate analysis in this study demonstrated a significant reduction in VAS scores following epidural morphine administration, particularly in women receiving a 2 mg dose. The large effect size underscores the efficacy of epidural opioids in managing acute postoperative pain, consistent with the established understanding of epidural morphine’s potent analgesic properties^[^20,21^]^.

Notably, the association between fasting adherence and lower VAS scores persisted after adjusting for epidural morphine dose (including the effective 2 mg regimen, consistent with prior evidence^[^20,21^]^), suggesting that fasting may exert effects beyond opioid-mediated analgesia. However, we caution against overinterpreting potential biological mechanisms. For instance, while some have hypothesized that shortened fasting might modulate inflammatory mediators or gastric stress responses, our study did not measure biomarkers such as IL-6, C-reactive protein (CRP), or cortisol, and any mechanistic claims remain speculative without direct empirical support.

### Influence of patient characteristics on pain outcomes

Stratified analyses revealed that certain patient characteristics significantly influenced the association between fasting duration and postoperative VAS scores. Notably, ASA III women, those with diabetes, hypertension, and higher intraoperative blood loss experienced greater reductions in VAS scores with extended fasting. These findings suggest that high-risk groups may benefit disproportionately from prolonged fasting protocols. In the stratified analysis of pregnant women with hypertension and diabetes, a significant decrease in postoperative VAS was observed for both drug combinations, warranting further investigation in subsequent studies^[^22^]^.

### Multiple regression analysis and clinical implications

Multiple regression analysis confirmed a significant correlation between ERAS fasting protocol and reduced acute postoperative VAS scores, even after adjusting for several covariates. Following full adjustment, which included morphine dosage, the significance of this association diminished, implying a consistent overall effect size when factoring in supplementary analgesic interventions.

Given its safety, low cost, and potential for synergistic benefit within multimodal care, adherence to ERAS fasting guidelines – particularly allowing clear fluids up to 2 h before surgery – should be routinely adopted in obstetric settings. Protocol standardization can reduce practice variability, enhance patient experience, and support broader ERAS compliance. Future quality improvement initiatives might audit fasting adherence as a process metric linked to recovery outcomes.

## Limitations of this study

This study has several limitations. First, as a single-center retrospective cohort, it is susceptible to selection bias, as women were not randomized to fasting groups and preoperative management may have been influenced by unrecorded clinical factors (e.g., urgency of delivery). Second, despite adjustment for key covariates, residual confounding remains possible due to unmeasured variables such as preoperative anxiety, pain catastrophizing, or intraoperative anesthetic variability – factors known to affect postoperative pain but not routinely documented. Third, data were derived from electronic medical records collected during routine care, which may be subject to documentation bias; for example, timing and accuracy of sensory block height or fasting duration could vary with clinical workload. Fourth, external validity is limited: findings reflect practice at a single tertiary obstetric center in China and may not generalize to other settings or populations. Finally, the study assessed only clinical outcomes (e.g., VAS scores, morphine use) and lacked mechanistic biomarkers (e.g., inflammatory cytokines or stress hormones) that could elucidate biological pathways linking fasting status to pain modulation. Despite these limitations, our large, representative cohort provides preliminary evidence supporting ERAS-compliant fasting as part of multimodal analgesia strategies in cesarean delivery women.

## Future directions

This study highlights the critical roles of preoperative fasting duration and multimodal analgesia in influencing postoperative pain outcomes in women. Understanding these relationships can inform evidence-based practices and enhance patient care quality in obstetric anesthesia. Future research should prioritize prospective, multicenter studies – ideally randomized controlled trials – to rigorously evaluate the impact of different preoperative fasting durations within Enhanced Recovery After Surgery (ERAS) pathways. Specifically, trials comparing shorter fasting intervals (e.g., ≤2 h for clear fluids and ≤6 h for light solids) against conventional or standard ERAS protocols would help clarify their effects on acute postoperative pain after cesarean delivery.

Moreover, future investigations should adopt a patient-centered outcome framework, integrating not only pain intensity (e.g., VAS scores) and analgesic consumption but also maternal satisfaction, time to ambulation, duration of urinary catheterization, length of hospital stay, and readmission rates. These recovery metrics are essential for assessing the holistic benefits of optimized fasting strategies in obstetric care.

Finally, incorporating biomarkers of inflammation and stress (e.g., IL-6, CRP, cortisol) alongside clinical outcomes could uncover potential physiological mechanisms linking gastric emptying, metabolic state, and pain perception – thereby strengthening the scientific foundation for evidence-based fasting guidelines in cesarean section.

## Conclusion

Adherence to ERAS fasting guidelines was associated with a clinically modest but statistically significant reduction in 24-h postoperative pain (β = −0.6, 95% CI: −0.9, −0.3; *P* < 0.001). Although the magnitude of pain reduction may be below the minimal clinically important difference, this non-pharmacological intervention − low-risk and easily implementable − supports its inclusion in standardized obstetric ERAS pathways and warrants consideration in future national and institutional guidelines.

## Data Availability

The datasets generated during and/or analyzed in the current study are not publicly available. However, data are available from the corresponding author upon reasonable request and with appropriate permissions.
